# Timely identification of palliative patients and anticipatory care planning by GPs: practical application of tools and a training programme

**DOI:** 10.1186/s12904-016-0112-9

**Published:** 2016-04-05

**Authors:** Bregje Thoonsen, Marieke Groot, Stans Verhagen, Chris van Weel, Kris Vissers, Yvonne Engels

**Affiliations:** Department of Anaesthesiology, Pain and Palliative Medicine, Radboud University Medical Center, Nijmegen, The Netherlands; Department of Primary and Community Care, Radboud University Medical Center, Nijmegen, The Netherlands; Department of Health Services Research and Quality, Australian National University, Canberra, Australia; Radboud University Medical Center, Post box 9101, Internal code 549, 6500 HB Nijmegen, The Netherlands

**Keywords:** Timely palliative care, Anticipatory, General practitioner, Training, Identification, Cancer, COPD, CHF

## Abstract

**Background:**

Palliative care is mainly restricted to terminal care. General practitioners (GPs) are not trained to early identify palliative patients with cancer, COPD or heart failure. With the help of the RADboud indicators for PAlliative Care needs (RADPAC), we trained GPs to identify patients’ needs and to make a proactive care plan. They were also able to join two role-plays where they discussed the patient’s future, and consulted a palliative care consultant to fine-tune the care plan. We evaluated the programme with the GPs and consultants and noted its impact on their daily practice.

**Methods:**

Two years after they had participated in the programme, we held semi-structured interviews with the GPs and a focus group interview with the consultants and performed a thematic content analysis.

**Results:**

Six consultants and nine GPs participated in the programme. Most GPs and consultants mentioned positive changes in the thinking or acting of GPs regarding early palliative care. A number continued to use the tool to identify patients; most of the others noted they had internalised the indicators. Although half of them still considered discussing end-of-life aspects difficult, particularly in patients with organ failure, the others were more easily able to discuss the future with their palliative patients.

**Conclusion:**

Although most GPs and consultants were positive about the training programme and applying it in daily practice, we conclude that in future programmes, more attention needs to paid to timely identification of palliative patients with COPD or CHF and how to discuss the future with them.

## Background

*Palliative care is an approach that improves the quality of life of patients and their families facing the problem associated with life-threatening illness, through the prevention and relief of suffering by means of early identification and impeccable assessment and treatment of pain and other problems, physical, psychosocial and spiritual* [1]. This widely accepted WHO definition shows that palliative care should not be restricted to reactive symptom relief and crises interventions. Timely palliative care makes it possible for the patient, the family caregiver and the healthcare professional to anticipate the wishes, future problems and terminal scenarios related to the patient’s situation. It improves quality of life and reduces depression and aggressive interventions in the last months of life [2–4]. However, early palliative care has not been widely implemented as the optimal starting moment, model, and structure are poorly defined.

In the Netherlands, most patients in their final stage of life live at home and would prefer to die there [5]. This implies that the general practitioner (GP) should be the coordinator of their care. Several studies report on the tools that have been developed to support the GP in this specific role: an instrument to help GPs stimulate patient-centred communication [6]; a method to improve interaction between GPs and district nurses [7]; and a communication training programme for GPs [8].

Dutch GPs have requested aids to help them timely identify their palliative patients. In response, we developed the Radboud indicators for PALliative Care needs (RADPAC) [9] (Appendix 1). In addition,, we developed the ‘problems square’ to help GPs make a structured, multidimensional overview of the patient’s current and future problems, needs, and advance care planning (ACP) wishes [10] (Appendix 2). We then trained GPs in identifying their palliative patients and in delivering structured proactive care. Following the training programme, the GPs were also offered a consultation by phone with a consultant specialized in palliative care for each identified patient. They were able to discuss the draft multidimensional care plan and the communication training with simulation patients [10]. We studied the effects in a randomised controlled trial (RCT) and found no differences between the intervention and control condition in number of contacts with the GP out-of-hours cooperative, hospitalizations, and place of death. Yet, the GPs only identified a quarter of all the palliative care patients who died in the year after the GP training programme. A post hoc analysis showed those patients identified by the trained GPs as needing palliative care, were 25 % less often hospitalized in the last three months of life, had more contacts with their GP (13 versus 7.5 contacts), more often died at home (67 % versus 45 %), and less often died in hospital (14 % versus 32 %) [11].

To gain better insights into the practical application of RADPAC and the training programme, we explored the views of both the GPs and the consultants who advised the GPs in order to fine-tune the proactive palliative care plan, two years after the GPs had been trained. We asked them to evaluate the tools, the model, and its application in daily practice.

## Methods

### Design

A qualitative study nested in the intervention condition of this larger RCT was conducted to get in-depth information of how participants evaluated the RCT’s tools and training programme [11]. We used a combination of focus group methods and individual telephone interviews [12].

### Ethical considerations

This study was part of a research project approved by the Research Ethics Committee of the Radboudumc (2007/205) in accordance with the Medical Research Involving Human Subjects Acts (WMO). It also conformed to the Helsinki Declaration [13]. Oral informed consent was obtained from all participants.

### Participants

Participants were (1) GPs who had participated in the RCT [11] and had been trained in timely palliative care two years prior to this study, and (2) consultants in palliative care who had specialised post-academic training in palliative care and with whom the GPs discussed the concept care plan. The main features of the intervention condition are described in Table 1.

**Table 1:** Intervention the trained GPs had received two year before

Two years prior to this qualitative study, the GPs that had been allocated to the intervention condition of a randomized controlled trial had received a five-hour group training in early identification and proactive palliative care planning. During this course, they received and practiced using the following tools developed by our research group: (1) the RADPAC, a tool with specific indicators to identify patients with COPD, CHF and cancer who might benefit from palliative care, and (2) a proactive palliative care planning card, the ‘Problems Square’: a tool designed to help users make a structured proactive care plan in which current and possible future problems (somatic; social and financial; caregiving and activities of daily living; and existential and psychological), dying scenarios, and patient’s wishes and needs are considered. The trained GPs were invited to apply this knowledge and these tools in their daily practice, and thus to identify palliative patients and to develop a proactive palliative care plan for each of them. With regards to each palliative patient the trained GPs identified, they were offered an individual coaching session by phone with a consultant specialised in palliative care. During this session the anticipatory care plan the GP had prepared was discussed, and adapted where needed. Finally, all trained GPs were offered two additional face-to-face peer group sessions in which experiences could be exchanged, and where they could practice with simulation patients to communicate end of life aspects. The GPs in the control condition had not received any training or intervention.	

### Focus group interview consultants

All consultants who had been consulted by any of the GPs trained in timely palliative care for a specific patient were invited to participate in a focus group interview. Each of them was asked to complete a questionnaire including demographic variables: gender, age, years of experience work as a palliative care consultant (fte – full time equivalent), and their other profession if they only worked as a consultant part-time.

An interview guide was developed based on the content of the intervention condition and extensive discussion within the project team. Using this guide, we explored: (a) experiences of the consultants in this new role as timely palliative care consultant; (b) the interaction with the GP during the consultations and the identification; and (c) the GP’s proactive care planning input. A GP moderated the focus group interview. Those consultants not able to join the focus group meeting were asked to participate in a semi-structured interview by phone.

### Telephone interviews with the trained GPs

We selected a convenience sample from the group of the 58 trained GPs and asked them to participate in a semi-structured telephone interview conducted by a trained female medical student. GPs were invited to participate and interviewed until saturation was reached.

The interviewer used a topic list, previously developed by the project team, to explore the GPs’ experiences with the training programme in general, identifying palliative patients, with the RADPAC identification tool [9] (Appendix 1) and their anticipatory care planning using the Problems Square (Appendix 2). After we received informed consent of each participant, both the focus group and individual telephone interviews were audio-recorded and transcribed verbatim.

### Data analysis

We used the software programme Atlas.ti 6 [14] for the (Dutch language) thematic content analysis. At first, we divided the transcripts into parts that covered the main aspects of the training and its application (see A through E, Table 2). Within each part, two researchers completed the line-by-line coding. They gradually discussed their codes until they reached consensus and the agreed codes were used in the consecutive transcripts; where possible, overarching themes were deduced. They compared the views from both the perspectives of the GPs and the consultants, if applicable. The deductive process, discrepancies and interpretations were regularly discussed within the research team.

**Table 2:** Description of themes and quotes

A The training
B Identification of the palliative patient
1. *“(…) regarding patients with CHF or COPD it becomes a hell of a job. And particularly when it concerns progress in time, that is also difficult. But when it concerns a patient with cancer, you know by and large (…) what is going to happen, and thus how to develop a plan.” (consultant)*
C Communication with the patient
2. *“But when you consider to start discussing end of life aspects, then I realize that such a message will come across the patient as quite a burden. […] (GP)*
3. *“I tended to communicate concealed and now I am more straight. (GP)*
4. *“It is possible to prepare patients, and I am better prepared myself too.” (GP)*
5. *“More than before, I keep an eye on them (…), people of whom you realize that they will not cure anymore, with whom it sooner or later will go wrong, like patients with COPD, CHF, that kind of people.” (GP)*
6. *“It is not just arranging more care, but also assessing what goes wrong or can go wrong and how can I anticipate on that (…) which can result in more care provision, but also in medication, or checking things.” (GP)*
D Proactive care planning with the problems square
7. *“I mean, as a GP I already did it that way but not as structured as with the problems square. And now it is easy to check did I consider all aspects, as it is sometimes mixed up.” (GP)*
8. *“I really liked the idea […] to consider prospectively what might occur […] in the several segments.”(GP)*
9. *“Doing it completely like stated in the protocol asks a lot of time, while many aspects will be addressed during the conversation anyhow.” (GP)*
10. *“With this kind of things, and that probably also counts for the problems square and the RADPAC, there will be a bigger chance that I use it when it is integrated as a protocol in the electronic medical record.”(GP)*
E Consultant – GP interaction
-Experiences of the consultant
11. “(…)*, as a consultant, you receive all kind of questions and honestly: I don’t know everything from neurology to paediatrics and all other specialties. So, also as a consultant you need dare to say: ‘I cannot answer this question, I will consult someone else myself (…).” (consultant)*
12. “*No, in fact nothing needed to be solved acutely. You could frankly take a helicopter view (…).” (consultant)*
-The role of the GP
13. *“The advantage is, (…) at the moment the GP fills in the problems square and he does it in a conscientious manner, than a lot of the potential future problems are already considered, just by filling in the form…” (consultant)*
14. *“The GP has a clear picture of the patient and probably knows him quite well. I, on the contrary, only know what has been filled in the problems square.” (consultant)*
-Interaction between consultant and GP
15. *“That is my trick, a bit as when you sit down to table (…) and give people room to talk, than you gain a lot more than when you just tick a check box.” (consultant)*
-Follow up
16. *“And at the same time, I would be curious whether the proactive suggestions you had provided (…) had landed and thus if the advice had an added value for the GP and probably for the palliative care for that specific patient…” (consultant)*

## Results

The group interview took 1.5 h. Four of the participating eight consultants worked part-time for one of the Dutch comprehensive cancer centres. Two of them were men and all were aged between 41 and 63. They had between 3 and 9 years’ experience as a palliative care consultant. One also worked as a GP, one as an elderly care physician, another as a palliative care consultant at a university medical center, and one as a teacher in palliative care courses. Two female consultants, a GP and an elderly care physician, were interviewed by phone as they had other commitments at the time of the focus group interview. The number of consultations per consultant performed within the prospective study varied between 1 and 11.

Of the thirteen GPs invited for interviews, nine were willing to participate; three women and six men. Saturation was reached after the seventh interview; during the 8 and 9th interview no new themes emerged.

The mean duration of the interviews was 17 min (5–25).

### GP evaluation of the training programme

Except for one, all GPs were positive about the training programme: they all mentioned positive changes in their thinking or acting as a result. For most GPs, this meant that their view on palliative care had changed: they were awakened to the fact that many of their chronic patients did not receive palliative care, although it was actually required. The GP who reacted less positively stated that he was already aware of what had been taught and practiced during the training programme.

### Identification of the palliative patient with the help of RADPAC

The respondents considered the indicators for timely identification of palliative patients with cancer, COPD or CHF to be clear [9]. Several GPs mentioned that they had integrated the indicators in their daily practice in such a way that they had them in mind and were able to use them directly, although others preferred to trust their clinical experience and evaluation of the patient. Several GPs still found timely recognition of palliative patients with organ failure difficult, despite using the indicators (Table 2, quote 1).

### Communication with the patient

When communicating with palliative patients with COPD or CHF, many GPs still found it difficult to discuss end of life aspects, as many patients do not realize that their condition is life-limiting and life threatening. (quote 2) With regard to communicating the transition from a merely curative to a palliative process with these patients, four GPs did not report any changes as a result of the programme. Two of them noted that they had never experienced any issues with these conversations, implying that the programme had not helped them. Most interviewees stated that they were more aware of, and paid greater attention to identifying patients with advanced stages of chronic conditions and who might benefit from palliative care, (quote 3), and several of them had the impression that communication with these patients had improved (quote 4). One of them mentioned paying more attention to future problems in the communication with his patients after being trained (quote 5). Some GPs observed real differences in how they predicted, communicated about and anticipated future problems; they discussed this more regularly and more proactively with their patients (quote 6).

### Proactive palliative care planning with the Problems Square

In general, the interviewees were positive about the Problems Square (Appendix 2). They stated that it helped them consider actual and possible future problems, needs and scenarios regarding all dimensions, and it prevented problems being overlooked (quotes 7–8). A few GPs still used the plasticized chart with the problems square, but most noted that they no longer needed it. The most important reason given for not using the plasticized version was time investment in relation to its added value (quote 9). At least three GPs would highly appreciate a digital version, integrated in the electronic medical record (quote 10). Another GP suggested adding the palliative care plan to the home care file in order to facilitate its use. One GP would appreciate an additional heading ‘euthanasia and palliative sedation’ in the format, stating that patients often have expectations and wishes regarding these topics, but in many cases neither the GP nor the patient raises them. One GP never used the proactive care plan at all.

### Consultant findings

#### Experiences of the consultants regarding proactive care planning

The consultants considered themselves capable of advising GPs on proactive care planning, however they also mentioned that their knowledge is limited and would like to have better insights into where they can find this missing knowledge (quote 11). They referred to elderly care physicians as being most likely to have expertise on palliative care for patients with CHF or COPD.

Most consultants agreed that a proactive care consultation takes more time than a standard consultation, however, when it is planned and not the result of a crisis, there is more time available (quote 12).

#### The role of the GP, as described by the consultants

Consultants appreciated the GP completing the problems square format prior to the consultation. In these cases, they noted that the GPs were better prepared and had better insights into the patient’s situation as well as the current and the potential future problems (quote 13). Yet, the accuracy and level of details when completing the problems square varied strongly. Often, only one or two ‘catchwords’ were noted which did not give the consultant a clear picture of the patient and context (quote 14); one consulted noted that the spiritual dimension was often left out.

#### Interaction between consultant and GP

Communication between the GP and consultant was considered to be valuable. The consultants considered themselves as sparring partners when discussing the scenarios and found it important to leave room for discussion, as this enabled them to dig deeper into the patient’s situation and give more detailed advice (quote 15).

#### Follow up

All consultants would have liked to have had a follow-up contact with the GP to explore which scenario had occurred and to hear what the GP had done with the consultant’s advice (Table 2, quote 16).

## Discussion

We evaluated the value of the GP training programme in identifying those patients that might profit from early palliative care, in communicating the future with the patient, and in structuring proactive palliative care. We also evaluated the use of the tools, the consultations, and how this influenced the care they provide.

Most GPs mentioned small changes in attitude and their way of thinking about palliative care and how to provide it. It widened their view on palliative care, and made them realize that patients with chronic diseases might also benefit from its timely initiation. These small changes are in line with the findings from two systematic reviews on educational interventions in the field of palliative care that show that education does have an effect on self-efficacy and attitude of physicians regarding palliative care, but only a limited effect on their daily behaviour [15, 16].

Although the RADPAC indicators that help GPs become aware of those patients that might benefit from palliative care were considered clear, most GPs no longer used the physical tool in their daily practice. However, several GPs stated that they had integrated the indicators in their daily practice. They also noted that they still found it difficult to recognize patients with organ failure who might benefit from palliative care. Other studies report that GPs and medical specialists experience barriers in palliative care provision to patients with COPD or CHF [17–19]; Dutch patients stated that clinicians rarely discussed life-sustaining treatment preferences, prognoses, dying processes, or spiritual issues with them [19]. Many GPs would appreciate a digital RADPAC tool, fully integrated in the electronic medical record system as has been developed in Scotland, where it was found to be successful in timely identifying palliative patients [20]. These findings are in line with the results of our RCT where only half of the trained GPs actually identified and reported patients who might benefit from palliative care. Of all the trained GPs’ patients with cancer, COPD or CHF who died in the year following the training programme, only one of four had been identified [11]. Of the patients who died, a third had COPD and/or CHF, while only 14 % had been identified as requiring pro-active palliative care. In a study on timely palliative care in patients with COPD [21], a clear and ‘natural’ moment to apply this type of tool was noted as being an event like acute hospitalization.

Some GPs still considered it difficult to start a conversation on anticipatory care with their patients with COPD or CHF. This is in line with a study by Janssen et al., which showed that most patients with COPD did not realize that they had a life-threatening disease, and that many of them do not feel that they are ready to talk about end-of-life issues [19]. In addition, the disease progress of COPD is often unpredictable, which can substantially hamper GPs in shared decision-making about future care options and needs [22]. Earlier studies report that barriers for end-of-life discussions with patients with organ failure appeared to be lack of time and lack of communication skills [19, 23]. Uncertainty about the course of the disease contributes to a ‘prognostic paralysis’, meaning that the GPs do not feel the urge to discuss end-of-life aspects with these patients. In contrast, most of the group of interviewees mentioned that it was currently easier to communicate, and that this better prepared them for proactive care planning in the future. Not all of the GPs interviewed joined the additional training sessions in which they could practice communication with simulation patients. We expect that those who had practiced with simulation patients who are in these kinds of conversations to feel more confident, as role-playing is an effective way of increasing communication techniques as well as doctors’ satisfaction about their communication skills [15, 24].

With regard to the proactive palliative care planning aspect of the training, most GPs stated that the Problems Square was valuable for structuring the inventory of actual and possible future problems, needs and wishes. This is in line with the findings of a questionnaire we sent to all GPs who participated in the trial, one year after the start of the RCT. Of the responding GPs, the trained GPs noted twice as often as the untrained GPs that they had conducted a multidimensional problems and needs assessment with the palliative patients they had at that moment (paper under review). Only a few still used the plasticized chart; the others said they had internalized it.

### Consultation

Although the consultants considered themselves capable of being a sparring partner for the GPs with regard to proactive palliative care planning, they were unable to answer each question. They stated that they needed to know where they could retrieve knowledge themselves if in doubt, or when they lacked the expertise; they also admitted needing extra training themselves with regard to proactive palliative care for patients with COPD or CHF. As we did not record the consultations between GPs and consultants, we cannot objectively evaluate how proactive the consultants’ advice was. The fact was noted that an anticipatory consultation takes them more time than a ‘normal’ consultation for acute problems in terminal patients. Yet, if anticipatory consultations are planned at a convenient moment, this extra time investment was not considered a problem, as it may even prevent acute consultations at a later stage.

The consultants were positive about the preparations the GPs had made prior to the consultation: the GP is the only person who can provide the complete picture of the patient, and the consultant is dependent on what the GP mentions. However, the consultants stated that the GPs only noted a few catchwords in the Problems Square, and often did not prepare the spiritual dimension. Our analysis shows that the combined spiritual/psychological dimension was only explored in about 40 % of the patients, but this was twice as often as the results of a large Dutch prospective study of untrained GPs.(paper under review). In this study, consultants also appeared to be better able to identify spiritual and psychological problems using problem clarification [25]. The EAPC taskforce on spiritual care in palliative care has the objective of improving this aspect of palliative care [26]. In the Netherlands, a nation-wide guideline on this subject has been published, of which an English translation is available [27]. Finally, the consultants would appreciate a systematically planned second consultation with the GP about the same patient, to evaluate the effects of their advice.

#### Strengths and weaknesses

This study provides insights into how those GPs trained in timely identification of patients in need of palliative care and in anticipatory care planning, perceived its value after two years. Although several studies have evaluated the effects of educational interventions on palliative care, the quality of those studies is often poor, and often restricted to short time effects [15, 16, 28]. With the help of the results of this qualitative study, future training modules in timely palliative care can be adapted to be more in line with GPs’ needs.

A strong point of this study is the triangulation: we interviewed both the GPs and the consultants. Studying a topic from different viewpoints gives a more complete image of the topic and increases its internal validity [29].

The study has a number of weaknesses. It became clear that many patients were under care of a hospital specialist who did not apply proactive palliative care. This means that the process of planning proactive palliative care is challenged; the GP misses natural trigger moments to apply RADPAC. Another fact was that our training programme did not provide an instruction on when to initiate joint primary-secondary care, or when to move back to primary care. This hindered identification and proactive care planning.

We used a convenience sample of GPs. In addition, we did not ask the GPs to evaluate the consultation, which means that this aspect of the intervention was only evaluated by the consultants. Given the range in the number of consultations held by the consultants, those with more consultations will have contributed more to the discussion than those who with less experience. We did not analyse whether participant demographics like age, years of experience or gender, influenced our findings.

As the interviews took place two years after the initial training sessions, it is likely that the GPs would not be aware of exactly what they had learned during the programme and what they had learned via other channels in the same period. Another potential weakness is that only four consultants took part in the focus group interview, although in the two phone interviews held after the group interview, no new information was revealed, indicating that saturation had been reached. Finally, we only interviewed a limited number of GPs and consultants, which means that we were unable to find differences regarding socio-demographic characteristics of the participating GPs and consultants.

## Conclusions

Two years after being trained in providing timely palliative care, GPs were still able to mention positive changes in their daily practice. They also noted difficulties initiating palliative care with their patients with organ failure, as they were still hesitant to discuss end-of-life aspects with these patients. This information has been used to optimise the training programme for GPs and consultants in this field. Aspects that required more attention in the training programme were (1) the use of a ‘natural’ marking moment such as information transfer between primary and secondary care or hospitalisation; (2) how to communicate end-of-life aspects with these patients; (3) To pay more attention to completing all the domains of the Problems Square, and (4) holding consultations with a palliative care expert to discuss and evaluate the proactive care plan.

Finally, we also recommend integrating identification and advance care planning tools in the GPs’ electronic medical record system.

## Figures and Tables

**Fig. 1:**
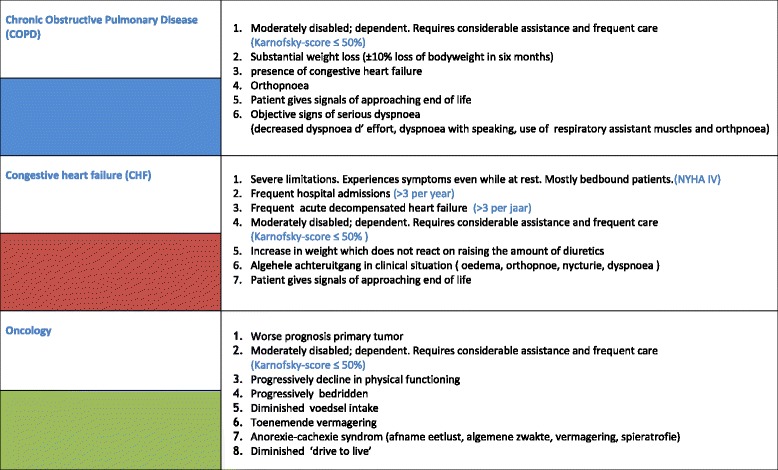
RADboud indicators for PAlliative Care needs (RADPAC)

**Fig. 2:**
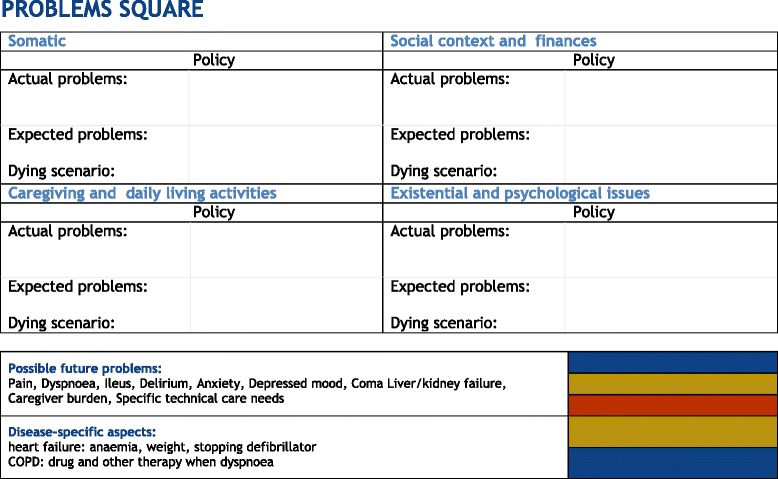
Problems square to make a structured actual and possible future multidimensional problems analysis

## Abbreviations

CHF: coronary heart failure
COPD: chronic obstructive pulmonary disease
EAPC: European Association for Palliative Care
GP: general practitioner
RADPAC: Radboud Indicators for Palliative Care Needs
